# Development obstacles of the agent accounting industry in China’s Guangdong-Hong Kong-Macao Greater Bay Area based on quantitative analysis—Research on the problem of employee’s work attitude

**DOI:** 10.3389/fpsyg.2022.930201

**Published:** 2022-09-12

**Authors:** Xiang Huang, Hyukku Lee, Mingyi Wang, Dong Wang, Yaoxian Wu, Kangsheng Du

**Affiliations:** ^1^School of Greater Bay Area Film and Television Industry, Guangdong University of Finance and Economics, Guangzhou, China; ^2^Department of East Asia Studies, Graduate School, Pai Chai University, Daejeon, South Korea; ^3^School of Application and Technology Institute, Hezhou University, Hezhou, China; ^4^Guangdong Polytechnic of Science and Technology, Guangzhou, China; ^5^School of Culture, Tourism and Geography, Guangdong University of Finance and Economics, Guangzhou, China

**Keywords:** agent bookkeeping company, emotional dimension, behavioral dimension, cognitive dimension, employee attitudes

## Abstract

The notion of “agent bookkeeping” was proposed when the “Accounting Law of the People’s Republic of China” was updated in 1993. Since their business is specialized in serving small and micro-enterprises, this has created the industry characteristic of generally small in the size of company and low in the salary of employees in Chinese agent bookkeeping companies. Such characteristic results in a series of problems including negative work attitude of employees in the development process, which seriously limit the development of Chinese agent bookkeeping companies. However, issues occurred in the development of agent bookkeeping, such as negative employee attitudes, which severely hampered the growth of Chinese agent bookkeeping companies. Therefore, a model of three dimensions of work attitude has been set up in this paper to demonstrate the working attitude of the staff of the agent bookkeeping companies in China’s Guangdong-Hong Kong-Macao Greater Bay Area, including emotional dimension, behavioral dimension, and cognitive dimension which consists of eight factors: work environment, career satisfaction, interpersonal relationship, role engagement, work vitality, responsibility attitude, emotional identity, and retention attitude. The result indicates that (1) female employees outnumber male ones in agent bookkeeping companies, (2) most employees have a low sense of belonging to the company, (3) limited career development affects employees’ enthusiasm for work, (4) work attitudes are influenced by mediocre work performance among employees with a graduate degree or higher.

## Introduction

China’s Guangdong-Hong Kong-Macao Greater Bay Area (GBA) is the world’s fourth-largest bay area, after the New York Bay Area, the San Francisco Bay Area, and the Tokyo Bay Area in Japan. It is a crucial special carrier for China to create a world-class urban agglomeration and compete on a global scale (China Government Network, 2019). China’s economy was affected by the COVID-19 pandemic, but it gradually recovered steadily. However, the problems of economical imbalance and insufficiency are also revealed. In particular, the recovery of small and micro-enterprises has lagged. Small and micro-enterprises are the driving force behind job creation and are closely linked to the life and development of China’s economy. Nonetheless, due to limited enterprise-scale and capital constraints, it is not likely for many small and micro-enterprises in the Greater Bay Area to establish an accounting department or recruit professional accounting personnel. According to Article 36 of the Accounting Law, in this circumstance, the corporation must entrust bookkeeping to an agent bookkeeping company (National People’s Congress Standing Committee, 2000). Hiring an agent bookkeeping company is not only less expensive than the salary cost of the accounting position, but it is also significantly less expensive than the service price charged by the accounting firm. The existence of agent bookkeeping companies solves the problems in the development of small and micro-enterprises, and it provides essential conditions and legal assurances for their growth and development.

According to the China Enterprise Credit Information Publicity System, there are now 374 agent bookkeeping companies in nine cities (excluding special administrative regions) in the Greater Bay Area. Resulting from the industrial and commercial department’s registration threshold limits, there are still many companies that run accounting businesses that did not register as agent bookkeeping companies when they go through the industrial and commercial registration. These companies provide bookkeeping services for small and micro-enterprises in the Greater Bay Area. According to the research, agent bookkeeping companies are often small in scale (around 5–15 employees), and overall low employee satisfaction leads to rapid staff loss, which is one of the key reasons why agency bookkeeping companies are difficult to develop. A questionnaire survey is conducted in this paper among workers in agent bookkeeping companies, followed by an interview with the person in charge. And then work attitude issues of the employees of the agent bookkeeping company were categorized and analyzed based on the analysis of the collected data as well as interview content, providing effective suggestions for the enterprise’s development.

## Research aims and research questions

The quality of the employees in agent bookkeeping company’s are widely varied, and the educational level is generally low with the absence of employee in senior professional post. What’s more, the proportion of intermediate professional employees to the total number of employees is low. The unreasonable structure of professional, too many non-professional personnel and insufficient education and training of employees lead to the overall negative working attitude of employees (Gu et al., 2021). An agent bookkeeping company is service-oriented that strives for customer satisfaction. If employees have a negative work attitude, customers will lose their trust in the company, and the business volume of the company will be difficult to expand or even shrink. Therefore, in the process of studying employees’ work attitudes, managers can gain a better understanding of the real situation of employees’ attitudes toward work, which can help the company correctly identify the differences in work attitudes among different employees and assist the company in classifying and managing employees using more effective management methods to achieve twice the result with half the effort.

## Definition and division of work attitudes

Employees’ positive or negative views of current occupations, as well as their proclivity to respond to actions, are referred to as job attitudes (Alexander and Freedman, 1984). Huang (1978) believed that an attitude is a collection of beliefs about a specific social object or social situation and that an attitude is formed through learning. Once an individual learns a certain attitude, it becomes a fairly persistent organization and can cause an individual to have a particular reaction tendency to the cognition, emotion, or action of the attitude object. Work attitudes, according to Robbins (1996), are made up of job satisfaction, work engagement, and organizational commitment. Rong (1998) believed that job satisfaction, organizational commitment, and work engagement are all key components of work attitudes. Hodgetts and Altman (1997) considered that attitudes comprise objective cognitive and behavioral elements as well as subjective emotional factors. Meyer (1993) proposed in the attitude ABC model that attitude could be classified into three aspects including emotional factor, behavioral factor, and cognitive factor. The emotion component primarily assesses employees’ feelings as well as their emotional experiences with the work and related personnel and things. According to the research of Cropanzano et al. (2002), the emotional dimension of work relates to employees’ feelings or emotional state at work. The behavioral dimension primarily shows individuals’ tendency of reaction at work. In the study of Schaufeli et al. (2002), the behavioral dimension (work engagement) should be divided into three factors: vitality, dedication, and concentration. Among them, vitality represents strong energy and psychological endurance and willingness to actively engage in work and solve difficulties. Dedication emphasizes the emotional experience of integrating high intensity into work. Concentration refers to focusing on work and working for a long time because it brings joy and pleasure to an individual. The cognitive dimension is primarily concerned with measuring employees’ recognition and evaluation of their work. Baltes et al. (2009) believed that organizational commitment means employees have a strong sense of identity and faith in the development direction and actual worth of the company so that employees are willing to make efforts to fit in to be part of the company. We combine the ideas from previous research, particularly the ABC model of attitudes, and Chinese researcher opinion of Zang and Zhu (2015), and classify accountants’ work attitudes into three dimensions which are emotional, cognitive, and behavioral aspects. These three dimensions consist of eight factors, which are working environment, career satisfaction, interpersonal relationship, role involvement, work vitality, responsibility attitude, emotional identity, and retention attitude (see Table 1).

**Table 1:** Dimension and factor division of work attitude.

	Dimension	Factor
Work attitude	Emotional dimension	Work environment
		Career satisfaction
		Interpersonal relationship
	behavioral dimension	Role engagement
		Work vitality
		Responsibility attitude
	Cognitive dimension	Emotional identity
		Retention attitude

## Materials and methods

### Quantitative study

The employees (accountants and their managers) of agent bookkeeping companies are the subjects in this study. Targeting this specific group of subjects, we reviewed a significant number of existing research results and refer to numerous work attitude subscales in China as well as overseas, such as the Minnesota Job Satisfaction Survey Scale, Utrecht Work Engagement Scale, and so on. Based on the work attitude scale of accounting personnel proposed by Professor Zang and Zhu (2015) and the full consideration of the research needs and actual situation of the field study in the Greater Bay Area, the questionnaire was revised and localized, and the initial work attitude questionnaire of accounting personnel was formed after expert discussion. After pre-testing, it was revised and perfected into a formal questionnaire. The questionnaire consists of three parts, the first part is the introduction. The second part is the measurement of the accounting staff’s working attitude, which is divided into three dimensions, namely emotional dimension, behavioral dimension, and cognitive dimension. The third part is the basic information, including age, duration of work, job title, position, educational background, and so on.

#### Questionnaire reliability and validity

Two hundred and twenty three questionnaires were distributed to agent bookkeeping companies in nine cities for this study, and 212 were retrieved. The sample questionnaire’s overall recovery rate was 95.06% and the number of valid questionnaires was 182, with an effective rate of 85.84%. This questionnaire’s overall scale of reliability coefficient is 0.966, which is more than 0.9. The coefficients of the three dimensions are all more than 0.9, while the coefficients of the eight factors are all more than 0.7. It completely demonstrates that the data is reliable and of good quality and that it can be used for further investigation (as seen in Table 2).

**Table 2:** The overall scale, the homogeneity coefficient of each dimension, and each factor.

Total table (45 items) α coefficient: 0.966					
Dimension	α coefficient	Factor	Number of questions	α coefficient	Reliability
Emotional dimension	0.947	Work environment	9	0.909	0.966
		Career satisfaction	4	0.874	
		Interpersonal relationship	3	0.893	
Behavioral dimension	0.943	Role engagement	7	0.874	
		Work vitality	6	0.879	
		Responsibility attitude	4	0.829	
Cognitive dimension	0.904	Emotional identity	6	0.781	
		Retention attitude	6	0.915	

The KMO value is 0.936, which is greater than 0.6, and the study items’ common degree values are all greater than 0.4, indicating that the information can be efficiently extracted. Finally, the variance explanation rate values for the seven components are 18.385%, 12.227%, 11.314%, 9.517%, 6.326%, 5.944%, and 5.111%, respectively, with the cumulative variance explanation rate after rotation being 68.824 > 50%. It signifies that the study item’s information can be properly extracted (as seen in Table 3).

**Table 3:** Validity analysis results.

	Factor loading coefficient						
	Basic question 1	Basic question 2	Basic question 3	Basic question 4	Basic question 5	Basic question 6	Basic question 7
Variance explained rate% (after rotation)	18.385%	12.227%	11.314%	9.517%	6.326%	5.944%	5.111%
KMO value	0.936						

### Interview

In this study, 11 interview questions were created using the Delphi approach. The interviews were question-based, with a focus on company operations, talent training, talent motivation, and talent recruitment. The person in charge of the agent bookkeeping company is the subject of the interview. The interviews were performed one-on-one and face-to-face. The interviews were all held in Chinese language with a total interview period of roughly 90 min.

## Results

### Descriptive analysis

This study employs SPSS analysis software to do descriptive statistical analysis on data from working period, professional titles, positions, educational background, and other industry variables. According to the study, women make up roughly 81% of the personnel at agent bookkeeping companies. Female employees accounted for 95% in some organizations in the field study, and the gender gap between men and women is significantly bigger than in other industries.

According to the study, 81% of respondents had “job-hopping” experience. Sixty seven percent of them have worked as accountants for more than 4 years. The majority which is approximately 67% of the employees had a low-level titles or no title at all. In terms of educational background, 95% of employees hold a college degree or higher. Last but not the least, only 24% of employees hold a CPA certification. Table 4 displays the descriptive statistics in detail.

**Table 4:** Descriptive statistics of variables.

		Frequency	Percent
Gender	Male	34	19.00
	Female	148	81.00
	Total	182	100.00
Number of units worked	1	34	19.00
	2–4	136	75.00
	>5	12	6.00
	Total	182	100.00
Working years	≤3	60	33.00
	4–10	92	51.00
	11–20	22	12.00
	≥21	8	4.00
	Total	182	100.00
job title	Assistant accountant	75	41.00
	Intermediate accountant	27	15.00
	Senior accountant	7	4.00
	None	51	28.00
	Other	22	12.00
	Total	182	100.00
Position	General accountant	139	76.00
	Accounting manager	43	24.00
	Total	182	100.00
Educational background	Graduate and above	9	5.00
	Undergraduate	113	62.00
	College	50	28.00
	High school/secondary school	8	4.00
	Other	2	1.00
	Total	182	100.00
Whether to obtain CPA certificate	Yes	43	24.00
	No	139	76.00
	Total	182	100.00

### Analysis of “job position”

The “job position” data was subjected to a *T*-test, and it was discovered that the *p*-values of numerous “test items” were less than 0.05, indicating that there was a significant difference in the variances of the two samples. Only the “test items” with the presence of significance (*p* < 0.05) are retained in the table of this study, and the rest are deleted. By summing the “test item” with *p*-values < 0.05, it is discovered that employees in various job portions have relatively big variances in their perspectives on role engagement, work vitality, and retention attitudes (Table 5).

**Table 5:** Analysis results of job *T*-test.

Dimension	Factor	Test item	Test item Job title (mean ± SD) P (job title)		P (position)
			General accountant	Accounting manager	
Emotional dimension	Work environment	4. I am satisfied with the salary and welfare system	2.72 ± 1.02	2.35 ± 1.02	0.039^*^
	Career satisfaction	11. When you perform well at work, you will be rewarded	2.68 ± 0.99	2.28 ± 1.08	0.023^*^
		12. I believe there are fair opportunities for promotion in the company	2.81 ± 1.05	2.28 ± 1.12	0.005^**^
Behavioral dimension	Role engagement	17. My work inspires me and brings me great satisfaction	2.45 ± 0.92	2.05 ± 0.87	0.013^*^
		18. The meaning of work is not only to meet my material needs, but also to meet my spiritual needs	2.51 ± 0.97	2.14 ± 1.04	0.033^*^
		19. My job is challenging for me	2.48 ± 0.95	2.07 ± 0.96	0.016^*^
		21. I feel happy when I work intensely	2.80 ± 0.97	2.33 ± 0.94	0.006^**^
	Work vitality	25. I can solve a lot of work problems	2.35 ± 0.78	2.05 ± 0.79	0.028^*^
		26. When I wake up in the morning, I want to go to work	3.05 ± 1.01	2.65 ± 1.09	0.027^*^
		27. I can work long hours at a time	2.58 ± 0.89	2.26 ± 0.95	0.040^*^
		29. I am passionate about my work	2.58 ± 0.91	2.26 ± 0.88	0.039^*^
	Responsibility attitude	31. I often advise companies on accounting treatment	2.68 ± 0.86	2.16 ± 0.87	0.001^**^
Cognitive dimension	Emotional identity	37. It is very difficult for me to leave the company at the moment	2.72 ± 0.96	2.35 ± 0.90	0.025^*^
	Retention attitude	39. There will be better room for development if you stay on the job	2.65 ± 0.93	2.23 ± 0.87	0.010^*^
		43. This job gives me a sense of security and stability	2.48 ± 0.98	2.09 ± 0.84	0.013^*^
		45. I am grateful for my current job	2.55 ± 0.98	2.09 ± 0.87	0.005^**^

* *p* < 0.05;

** *p* < 0.01.

1. When comparing the average value of the “role engagement” component, “general accountant” is more likely than “accounting manager” to choose “disagree” for “test items 17, 18, 19, 21,” showing that individuals in the lower rank of position are more likely to choose “disagree.” Employees are less spiritually pleased with work, and it is more difficult for them to devote themselves to their jobs.
2. When comparing the average value of the “work vitality” component, “general accountant” is more likely than “accounting manager” to choose “disagree” for “test items 25, 26, 27, 29,” showing that individuals in lower rank of position are more likely to choose “disagree.” Employees are less passionate and active, and they are less motivated to address difficulties.
3. When comparing the average value of the factor “retention attitude,” “general accountant” is more likely than “accounting manager” to select “disagree” for “test items 39, 43, 45,” indicating employees in lower rank of position lack job security. They believe that there is no room for promotion in their current position.

To sum up, employees in the low rank position believe that their opportunities for promotion in current company are restricted and limited, which affects their motivation for work.

### Analysis of “educational background”

**Figure 1:**
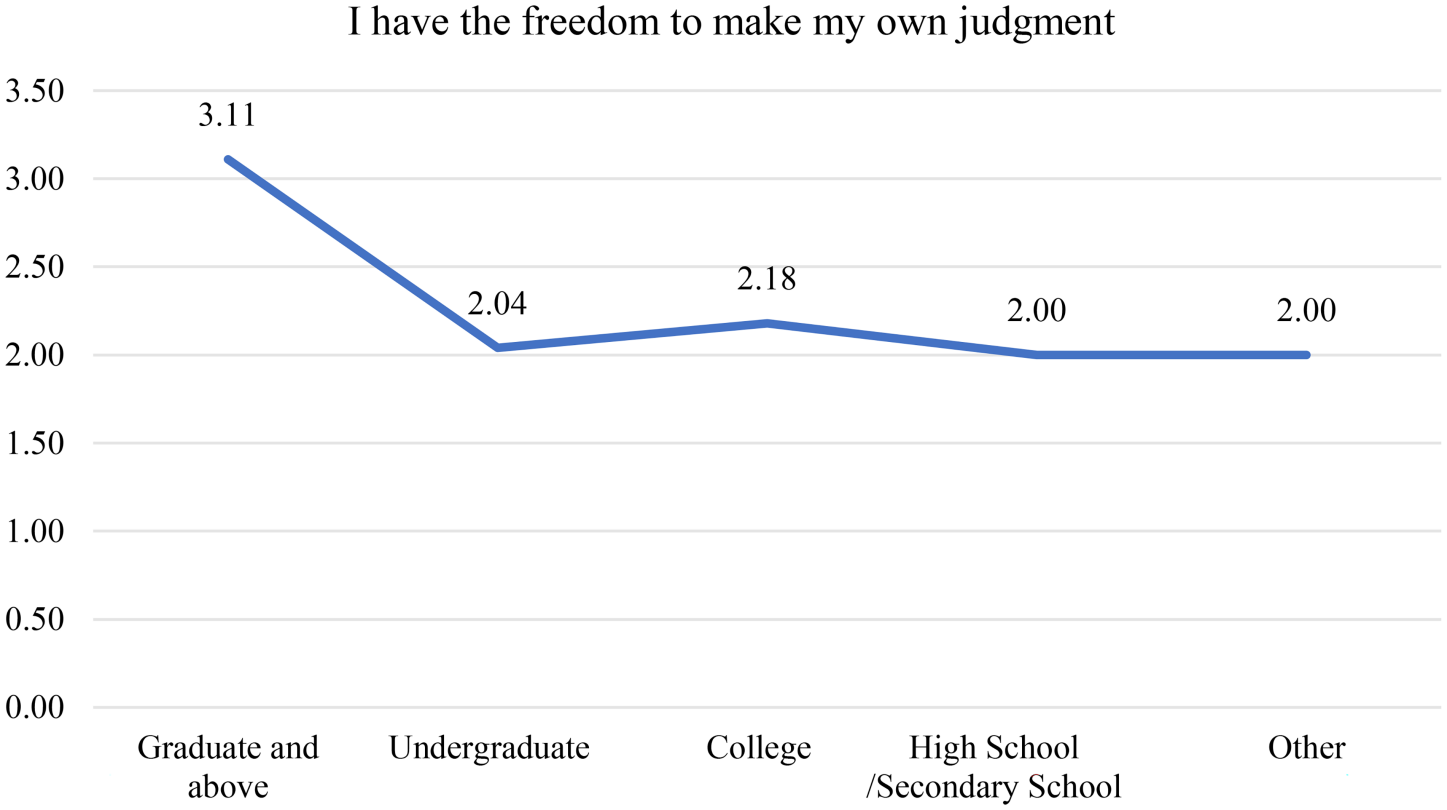
Analysis of variance for the question “I have the freedom to make my own judgments.”

The findings of the analysis of variance for the question “I have the freedom to make my own judgement” and the results of the chi-square analysis of the educational background and work titles are shown, respectively, in Figure 1 and Table 6. Employees with a “graduate and above” degree tended to select “Uncertain, “while those with a “Undergraduate or lower” degree tended to select “Agree.” The distinction between the two groups is evident. Although individuals with a higher academic degree are unable to make independent decisions at work, which is unreasonable. Therefore, we must examine the cross-examination findings for “educational background” and “job title” in Table 6. There are 9 personnel with a graduate degree or higher, of which 3 are “management accountants” and 6 are “general accountants.” With an analysis of the original data, it was discovered that the majority of the 6 “general accountants” with a doctoral degree or higher chose “disagree” or “uncertain” on the question “I have the freedom to make my own judgement.” In the interview with the agent bookkeeping company’s manager, it was discovered that these six employees with a graduate degree or higher had typically mediocre work performance and low level of initiative, because they failed to be employed by “accounting firms” or “major enterprises” and ended up working for lower-paying bookkeeping firms.

**Table 6:** The results of the cross (Chi-square) analysis of educational background and job title.

Title	Name	Your position is		Total	Value of *p*
		General accountant	Management accountant		
Your highest education is	Graduate and above	6 (4.32)	3 (6.98)	9 (4.95)	0.455>0.05
	Undergraduate	85 (61.15)	28 (65.12)	113 (62.09)	
	College	38 (27.34)	12 (27.91)	50 (27.47)	
	High school /secondary school	8 (5.76)	0 (0.00)	8 (4.40)	
	Other	2 (1.44)	0 (0.00)	2 (1.10)	
Total		139	43	182	

### Analysis of interviews

In the interview with the managers of the agent bookkeeping company, it concludes that:

1. All general accountants with a graduate or higher degree are new employees with little experience.
2. Generally speaking, general accountants with a graduate or higher degree have mediocre working competence and poor performance in communication skills.
3. General accountants with a graduate or higher degree were rejected by “accounting firms” and “major enterprises,” then they were left with nothing better than the second choice, which is lower-paying agent bookkeeping companies.
4. Due to the high turnover of personnel, the person in charge of the agent bookkeeping company does not pay enough attention to the employees’ work attitudes.

In the interview with the general accountants of the agent bookkeeping company, it concludes that:

1. The male-to-female ratio of the agent bookkeeping company’s personnel is similar to the male-to-female ratio of accounting majors in school.
2. Male employees often believe that agent bookkeeping firms provide lesser wages. And in traditional Chinese conceptions, males have to meet somewhat larger economic demands, thus men are more hesitant to enter this industry.
3. In general, females outnumber males in agent bookkeeping firms. Female employees have too few opportunities to meet an appropriate date at work, and they are unable to find proper spouses at the appropriate age, resulting in issues such as high employee mobility.

## Conclusion

### Conclusion of the investigation

**Figure 2:**
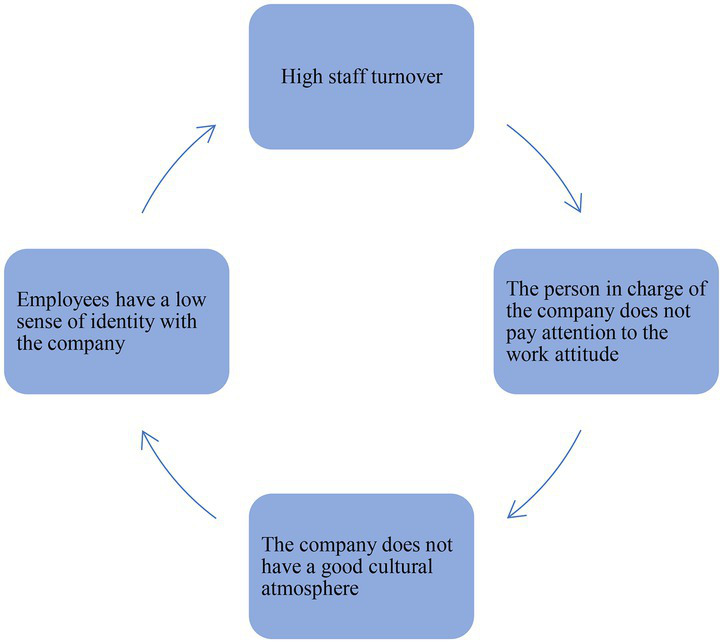
Staff turnover circle structure.

**Figure 3:**
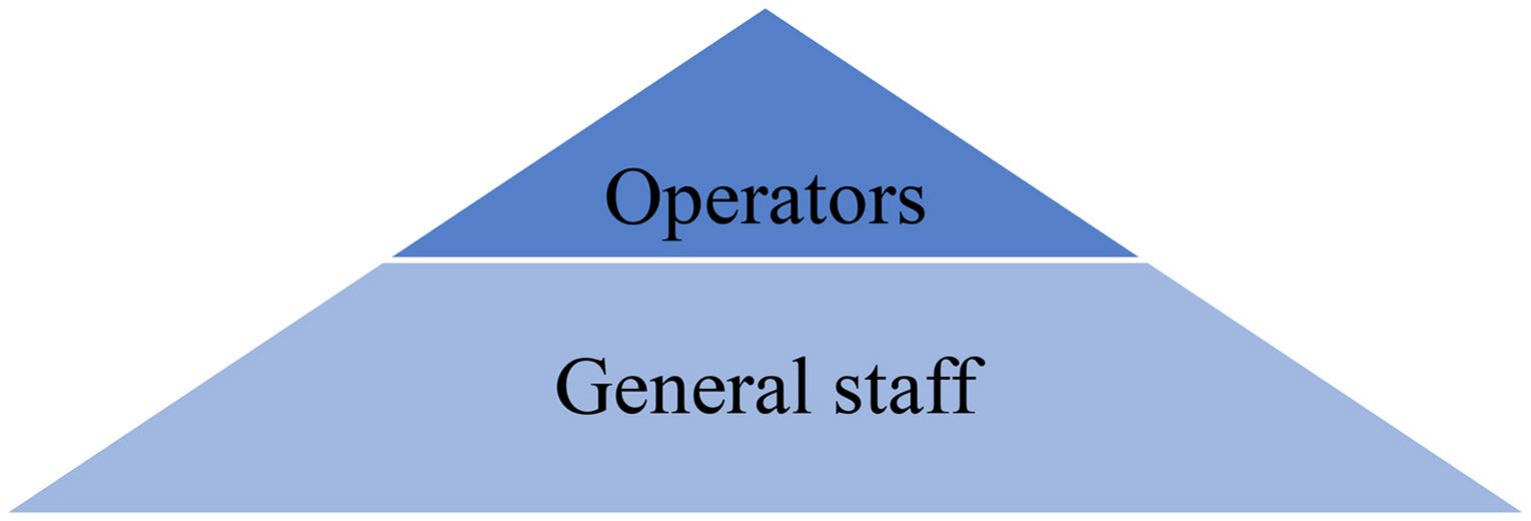
Agent bookkeeping companies’ personnel structure.

According to the above analysis of the overall situation and classification of the work attitudes of employees of agent bookkeeping companies in the Greater Bay Area, the main factors affecting the work attitudes of employees of agent bookkeeping companies in the Greater Bay Area include work environment, career satisfaction, role engagement, work vitality, responsibility attitude, emotional identity, and retention attitude. To summarize, the concerns identified in this study are primarily comprised of the four aspects listed below:

1. In general, there are more women than men in agent bookkeeping companies. Female employees outnumber male employees in agent bookkeeping companies, and the gender disparity between males and females is significantly greater than in other industries. The interview analysis highlighted the reasons for this phenomenon.
*2. Employees have a weak sense of belonging to the company.* The employee turnover rate is relatively high, and employees have a low sense of identity within the company. This kind of issue arises due to two reasons:
(1) The salary system is inflexible. The amount of work is out of proportion to the salary, and employees find it difficult to achieve more wages. A good salary is one of the most effective techniques for motivating employees. It can have a good impact on employees and motivate them to overcome obstacles at work. When the salary system of a company restricts compensation for employees’ work and fail to motivate them at work, it is likely to induce opportunistic behaviors, and the repercussions are reflected in company investment, which may indicate low investment efficiency (Liu and Tai, 2020). Fix salary influences employees’ passion for their work inevitably.
(2) There is no emotional attachment to the company. A modern enterprise, even a private firm, needs cohesive employees rather than authoritarian leadership. Not just some but every employee should be involved in the development of business culture (Cheng, 2020). It was discovered through interviews that the company leaders generally did not pay enough attention to the employees’ work attitudes. What is more, agent bookkeeping companies are typically small in size, have little capital, and have high employee turnover. These are the reasons causing a lack of a good cultural atmosphere in the company, and the employees would eventually have a low sense of identity with the company, resulting in the problem of high employee turnover in the company. This is a never-ending vicious circle (Figure 2).

3. *Limited career development has an impact on employees’ work enthusiasm.* The study discovered that the level of job title influences work attitude. And the overall work attitude of employees in agent bookkeeping companies is poor, according to the aforementioned significant difference analysis. Whether measured in the perspective of “career satisfaction,” “role engagement,” “work vitality,” or “responsibility attitude,” most employees pick “dissatisfaction,” indicating that their total job satisfaction is low. For grass-root employees, the lack of adequate promotion space will damage their enthusiasm for work. The design of an employee career development channel is the only way for any company to encourage employee growth and improve internal management (Ji and Yang, 2015). However, the majority of agent bookkeeping companies in the Greater Bay Area are small or micro-enterprises with about 5–15 employees. The personnel structure mostly adopts the model of “operator plus ordinary staff.” (Figure 3). Hence The company needs to implement effective employee career development policies to compensate for the limitation of employee promotion.

*4. Mediocre work performance of employees with a graduate or higher degree often influences their work attitude.* There are instances where highly educated employees have mediocre performance on their job. They were rejected by large corporations or accounting firms, and agent bookkeeping companies are their second best choice. We learned from the conversations with the person in charge that these kinds of employees have relatively low ability to complete work independently and solve problems. Spitzberg and Cupach (2012) defined social communication competency as “A person’s ability to interact effectively with others.” According to Sager et al. (2015), the ability to adapt to interpersonal communication promotes constructive patterns of interaction. To get the job done in the bookkeeping sector, employees must be able to communicate effectively with customers. Interviews with employees with a graduate or higher degree revealed that they are mostly introverted and have poor socializing and interpersonal skills, resulting in poor work attitudes.

### Innovation

1. Due to the special tax policy and accounting system in China, the legal procedures for the registration of agent bookkeeping companies are more complicated, and many agent bookkeeping companies are not registered as agent bookkeeping companies but are registered as other company types and continue to operate agent bookkeeping business, and the number of this type of agent bookkeeping companies is high. Most of the previous studies have not covered this situation, but this study includes it in the scope of the study.
2. There are many papers on work attitudes and many studies on accountants regarding academic accounting issues, but there are almost no studies on the work attitudes of accountants in the special industry of agent bookkeeping.

### Limitations

1. Since many agent bookkeeping companies are not registered as agent bookkeeping companies at the time of registration due to the special policy factors mentioned earlier, this study cannot identify all such companies, and the scope of the study is somewhat limited.
2. A small number of companies that were not registered as agent bookkeeping companies at the time of registration but operated agent bookkeeping businesses were not willing to be interviewed and researched due to the fear of legal issues, so the scope of the study has some limitations.

## Suggestion

1. *Develop a “Mentoring System” Training Program.* According to Wassem et al. (2019), skill training for employees enhances organizational efficiency. Employees’ work attitude can be improved with sufficient training, allowing them to fully utilize their talents at work and benefit the company. This study advises using a “mentoring system” training plan to train the new employees. Leaders or senior employees train the first group of employees. After a time of tempering and follow-up instruction, the majority of these employees can eventually gain enough experience to be able to finish their tasks independently and solve problems by themselves. And then they become mentors who can train the next group of new employees. They can assist and instruct new employees as well as other employees without interfering with their duties. Employees’ general job ability and work attitudes can be improved gradually by using the “mentoring system” strategy.
2. *Revise the recruitment requirement. A graduate or higher degree should not be a prerequisite.* Educational background is a person’s academic experience, while ability is a person’s capacity to perform things (Tang, 2014). The field study discovered that the professional requirements of work of bookkeeping companies are less than that of accounting firms, thus a high academic degree should not be a priority in recruitment. Employees with strong initiative are what the agent bookkeeping company needs the most. Employees are just required to perform basic and mechanical accounting and bookkeeping services. Undergraduates and college students are fully qualified for the work. As a result, the focus of recruitment should be on employees’ subjective initiative and communication skills. Employees with a graduate or higher degree are more dissatisfied with low wages and limited career growth in agent bookkeeping companies, according to the study. And emotions such as dissatisfaction can be contagious among employees. Therefore, a high level of staff turnover may be resulting from hiring individuals with a graduate or higher degree.
3. *Establish a “learning-oriented” and “home-like” company culture to decrease employee turnover.* Advanced company culture is the spiritual driving force and an important guarantee of the enterprise’s cohesion, reform and development (Qian, 2020). Through interviews and data analysis, the study discovered that agent bookkeeping companies, in general, do not pay attention to company culture establishment. The reason for this is that these companies are small in size and employ a small number of people. Most operators see no need to spend time and money on it. The personnel of an agent bookkeeping company lacks centripetal cohesion when compared to that of a corporation with a company culture. Aiming at the contemporary post-90s and post-00s employees with strong subjective consciousness, small and micro-enterprises such as an agent bookkeeping company should establish a company culture to improve employee morale. Since the majority of agent bookkeeping companies are small and micro companies with limited scale and capital, they cannot significantly raise wages or benefits, whereas it takes not much work to establish a company culture. (1) Through regular “team building,” the company can establish a “home-like” culture. Managers of the company act as “parents” to care about the physical and mental health of employees. Employees have a good emotional contact with the organization. With a good emotional interaction between employees and the company, employees can naturally increase their sense of identity with the company. (2) By organizing small group training, such as accounting skill training in different industries, industrial and commercial business training, junior and intermediate accountant training, etc., “learning-oriented” company culture is established. Continuous learning is a never ending force of motivation for career development. On one hand, in terms of life-long learning, it is important to update knowledge and acquire new abilities continuous to increase one’s career development potential. On the other hand, it can boost one’s confidence and gain more sense of assertiveness (Wang, 2021). Therefore, organizing “small group” training not only improves the professional ability of employees, but also promotes the spirit of learning from each other. Employees can accept company’s cultivation and attention to them at the same time. Many of the operators of agent bookkeeping companies we interviewed agreed with this suggestion and are ready to implement it.
4. *Implement “decentralization,” “profit sharing” and “honor incentive mechanism” mechanisms.* Employees have different levels of work competencies as well as different levels of job efficiency. If employees with a higher level of abilities are paid the same as those with lower level of abilities, they will feel unfair and become more and more dissatisfied with the company. This study discovered through interviews and investigations that agent bookkeeping companies are often small in scale, with less capital and high personnel mobility. The head of the company does not pay attention to individual employees and usually overlooks individual employee’s work efficiency. A fixed salary payment system is adopted with such extensive management. According to this report:

First, it is necessary to implement a “profit sharing” mechanism which is the “basic” and “performance” salaries combined. The performance salary is based on commission. The employee takes commission from set proportion of the service fee paid by the customer. More customers, more commission. The Employee gets more commission from a big client than a smaller one. After the companies tried implementing a “profit-sharing” system based on our suggestion, employees’ passion for work raised rapidly. Some employees even returned to the company to work overtime voluntarily.

Second, the “decentralization” mechanism is also essential. Because of the limited number of employees and the high volume of business, agent bookkeeping companies simply assign the same amount of work to each employee. Nonetheless, work should be gained but not given. Besides, there are some employees with good ability who are willing to work more. The company can give such employees the right to find and choose customers by themselves and let them be in charge of the business of big clients independently. When the company implements the “performance” salary payment system, such top performers can earn more than others. The superior employee will naturally be selected to stay and the inferior will be eliminated in the end. Hence the company will be left with elites with superior work capacity and great work attitude.

Third, it is important to apply the “honor incentive mechanism” as a motivation booster. Many agent bookkeeping companies are small in scale and often neglect the contributions made by excellent employees to the company and do not appreciate their work, which causes employees to lose motivation and confidence in their job. We suggest the company reward employees by giving out awards such as “Employee of the Year,” “Accounting Master,” “Most Favoured Accountant,” as well as appropriate bonuses according to their work performance.

5. *Last but not the least, agent bookkeeping companies could organize a variety of activities to encourage employees to interact with people who could be potential date or potential client.* In general, agent bookkeeping companies have fewer employees than other companies and more women than men, making it difficult for employees, particularly female employees to find appropriate date, resulting in high turnover rate. In order to solve such conundrum, the company could encourage employees to participate in more group training, such as tax training offered by the tax bureau and accounting training offered by the finance bureau, so that they can not only meet new friends, but also learn professional knowledge, effectively killing two birds with one stone. In addition, agent bookkeeping companies could organize social events such as networking cocktail party with client companies. Employees could broaden social network as well as retain client interactions. It counts as one kind of company team building events to improve employee morale as well. Thirdly, agent bookkeeping companies could encourage employees to participate in more outdoor activities to meet new people. Employees could also develop new clients when participating in outdoor activities. And employees who successfully sign new contract with new clients will be rewarded in terms of monthly bonus. In this way, employees would be more interested in stepping outside of the box to meet with new people.
6. *Agent bookkeeping companies could also provide opportunities for training and help employees to enhance their social skills.* To cope with the situation where employees with a graduate or higher degree have mediocre work performance, we suggest managers of agent bookkeeping companies pay more attention to the needs of such employees and try to place them in appropriate positions where they could shine because they are more interested in or skilled with. For example, employees who are interested in bookkeeping could be placed in accounting positions. Second, such employees usually lack social skills and are afraid of making mistakes when communicating with customers, hence they often avoid dealing with customers, resulting in negative work attitude. Avoiding communication with clients also eliminates the opportunity for them to improve social skills. It could be a vicious circle. Agent bookkeeping companies could provide opportunities, such as organizing speech contest, for employees to demonstrate speech to gain more eloquence and boost their confident to overcoming Social Phobia by requiring each employee to report on their work at the weekly/monthly/yearly meeting.

## Data availability statement

The original contributions presented in the study are included in the article/supplementary material, further inquiries can be directed to the corresponding author.

## Ethics statement

Ethical review and approval was not required for the study on human participants in accordance with the local legislation and institutional requirements. Written informed consent from the patients/participants or patients/participants legal guardian/next of kin was not required to participate in this study in accordance with the national legislation and the institutional requirements.

## Author contributions

MW: conceptualization and writing—review and editing. XH: methodology and writing—original draft. HL: conceptualization and data curation. DW: data curation. YW: translation. KD: investigation. All authors contributed to the article and approved the submitted version.

## Funding

This research is financed by the Guangdong Provincial Education Department (No. 2019WTSCX031).

## Conflict of interest

The authors declare that the research was conducted in the absence of any commercial or financial relationships that could be construed as a potential conflict of interest.

## Publisher’s note

All claims expressed in this article are solely those of the authors and do not necessarily represent those of their affiliated organizations, or those of the publisher, the editors and the reviewers. Any product that may be evaluated in this article, or claim that may be made by its manufacturer, is not guaranteed or endorsed by the publisher.
